# Supplementary material for: A methodology for the design of experiments in computational intelligence with multiple regression models
Source: PeerJ. 2016 Dec 1;4:e2721. doi: 10.7717/peerj.2721 (PMC5136129; doi:10.7717/peerj.2721)
Supplement: Data S1 — Datailed results from UC Irvine Machine Learning Repository (Housing, Machine CPU, Wine Quality, Automobile and Parkinson) and the 3 Use Cases (Protein Corona, Gajewicz Metal Oxides and Aquatic Toxicity) [file peerj-04-2721-s001.zip › 6_UseCases/3_AquaticToxicity/GLM.details.csv.repeatedcv.split9.pdf]

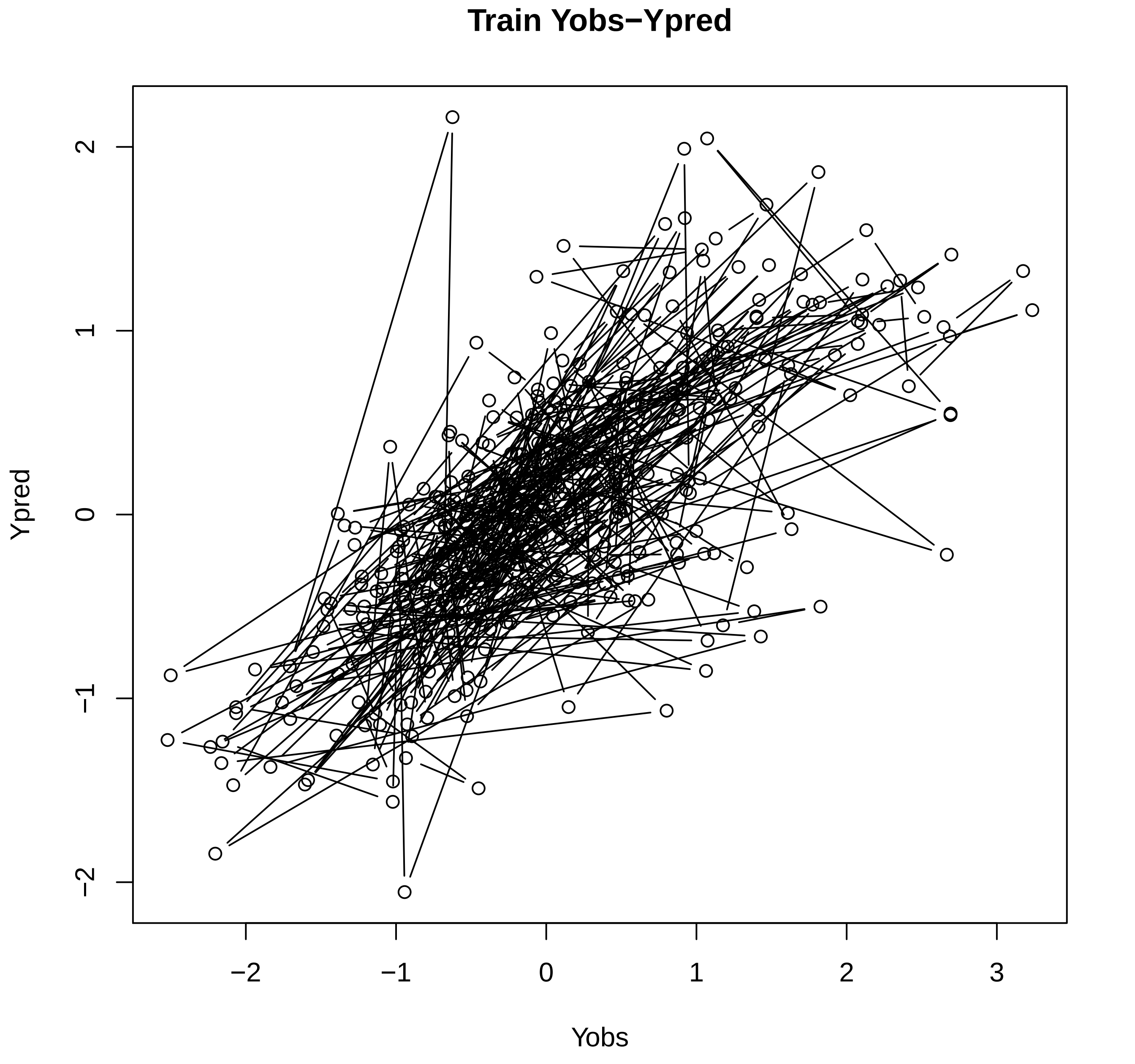
Train Yobs−Ypred
Ypred
Yobs
2
1
0
−1
−2
−2
−1
0
1
2
3

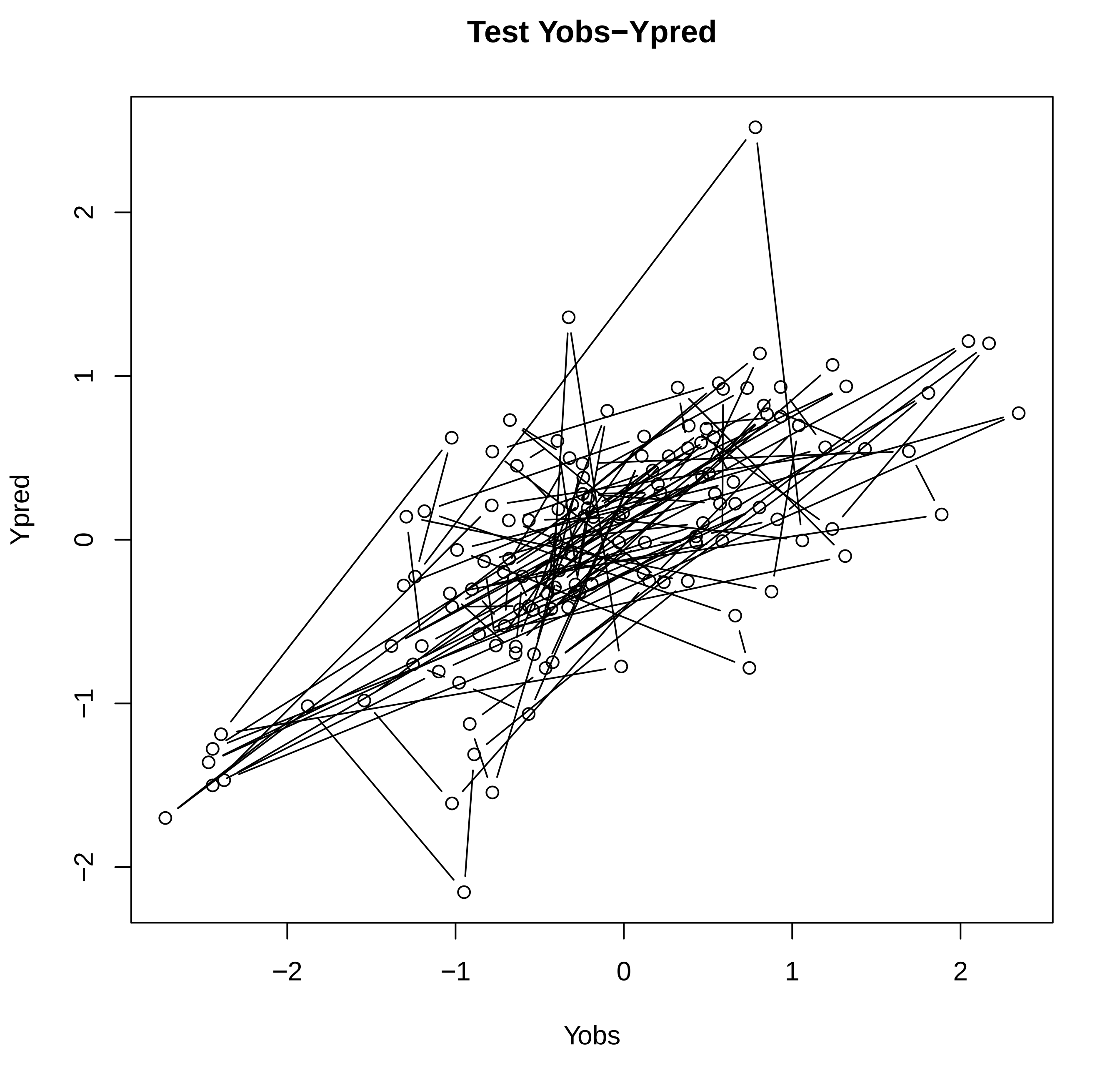
Test Yobs−Ypred
Ypred
Yobs
−2
−1
0
1
2
−2
−1
0
1
2

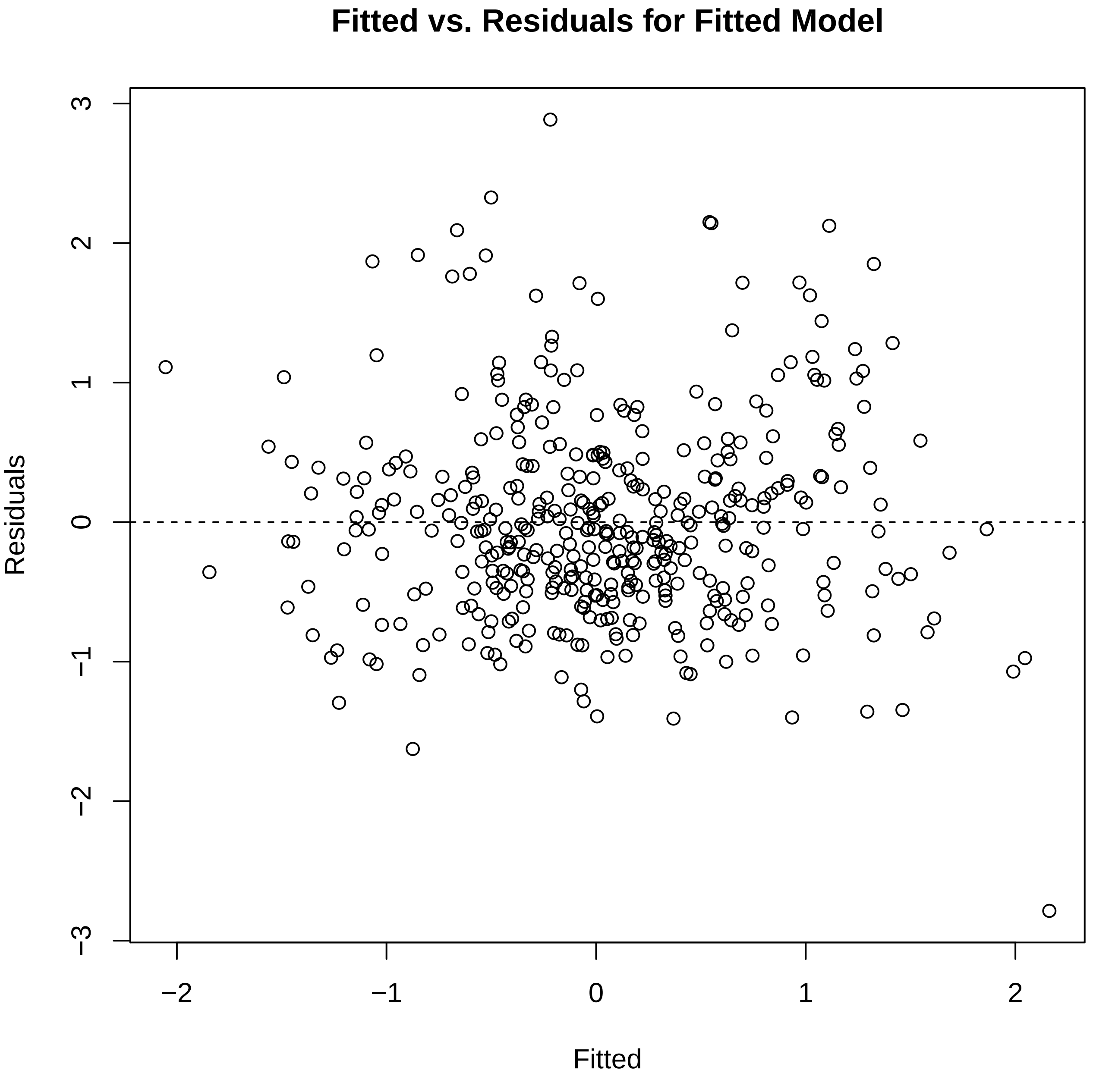
Fitted vs. Residuals for Fitted Model
Residuals
Fitted
3
2
1
0
−1
−2
−3
−2
−1
0
1
2

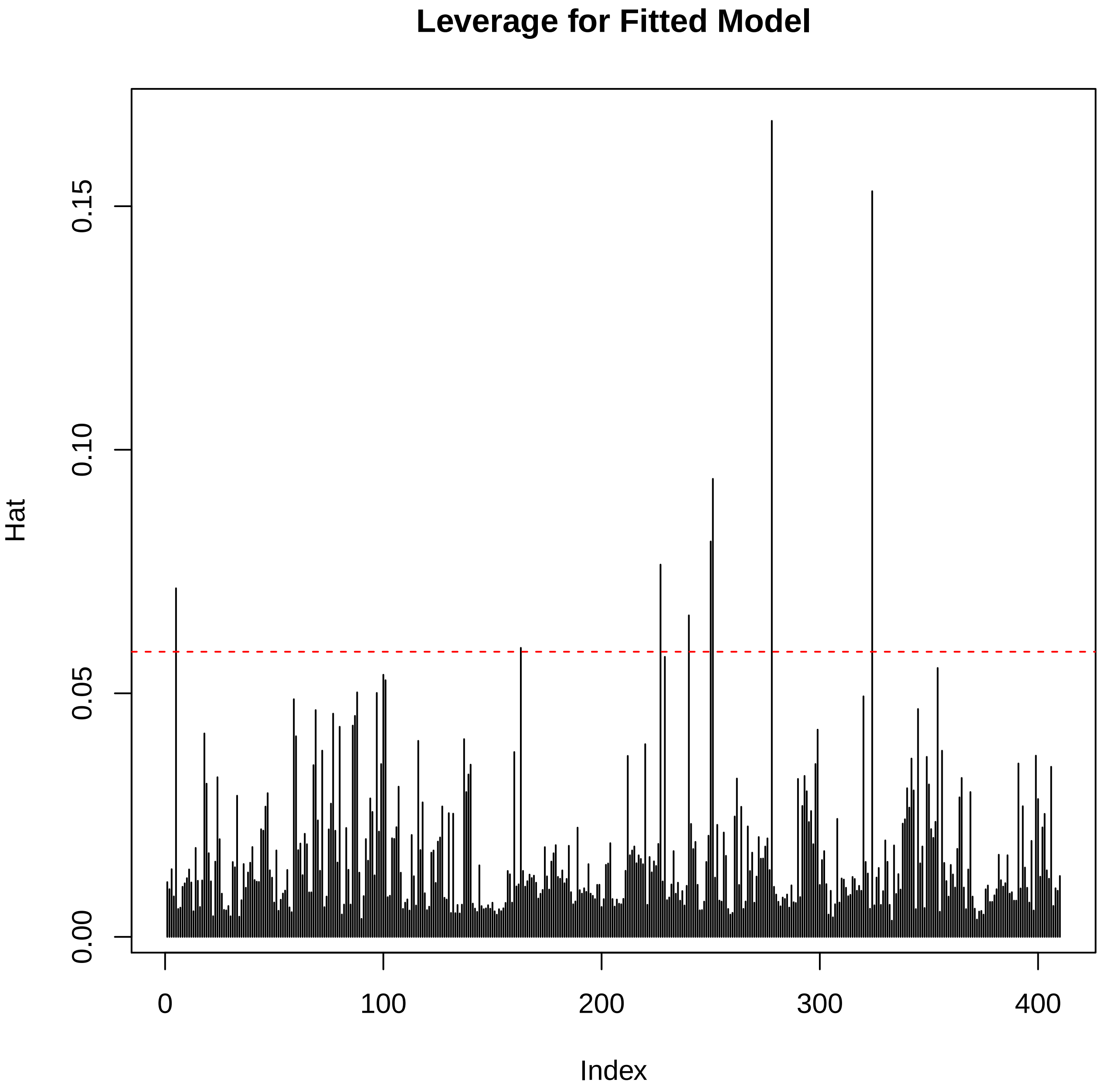
Leverage for Fitted Model
Hat
0.00
0.05
0.10
0.15
Index
0
100
200
300
400
